# Two new species of the genus *Asceua* Thorell, 1887 (Araneae, Zodariidae) from China

**DOI:** 10.3897/BDJ.11.e103298

**Published:** 2023-04-11

**Authors:** Wei Wang, Yejie Lin, Xiaoqing Zhang, Chang Chu, Shuqiang Li, Chengming Huang

**Affiliations:** 1 Guangxi Key Laboratory of Rare and Endangered Animal Ecology, College of Life Sciences, Guangxi Normal University, Guilin, Guangxi 541006, China Guangxi Key Laboratory of Rare and Endangered Animal Ecology, College of Life Sciences, Guangxi Normal University Guilin, Guangxi 541006 China; 2 Hebei Key Laboratory of Animal Diversity, College of Life Science, Langfang Normal University, Langfang, Hebei 065000, China Hebei Key Laboratory of Animal Diversity, College of Life Science, Langfang Normal University Langfang, Hebei 065000 China; 3 Institute of Zoology, Chinese Academy of Sciences, Beijing 100101, China Institute of Zoology, Chinese Academy of Sciences Beijing 100101 China; 4 College of Life Science, Shenyang Normal University, Shenyang, Liaoning 110034, China College of Life Science, Shenyang Normal University Shenyang, Liaoning 110034 China

**Keywords:** Hainan, Jiangsu, taxonomy, diagnosis, type

## Abstract

**Background:**

The spider genus *Asceua* Thorell, 1887 contains 34 species, almost entirely limited to Indochina, India, Sri Lanka and China, with a regional distribution. Eleven species of *Asceua* are currently only known from China, five of them are described only from one sex.

**New information:**

Two new spider species of the genus *Asceua* are reported from China, *A.haocongi* sp. n. (♂♀, Hainan) and *A.zijin* sp. n. (♂♀, Jiangsu). Photos and a morphological description of the new species are provided.

## Introduction

The ant spider family Zodariidae Thorell, 1881 contains 90 genera and 1264 known species worldwide (World Spider Catalog 2023). Members of this family are small to medium-sized (Jocqué 1991). *Asceua* Thorell, 1887, with the type species *A.elegans* Thorell, 1887 described from Myanmar, is a relatively large genus of the family Zodariidae and currently comprises 34 species mainly distributed in Asia and Africa. Members of this genus can be distinguished from other zodariids by their small size, laterally compressed bulb, developed cymbial fold and long and meandering copulatory ducts (Zhang and Zhang 2018). Recently, a large number of new spider species have been reported from China (Li 2020, Li et al. 2021, Yao et al. 2021, Zhao et al. 2022, Lu et al. 2022), but Zodariidae is poorly studied in China, especially for Asceua. Until now, only 11 species are known, all species are endemic to China except, *A.torquata* (Simon, 1909) from China, Laos and Vietnam, most of those being distributed in Yunnan and Hainan (Song and Kim 1997, Yin et al. 2012, Zhang et al. 2012, Barrion et al. 2013, Zhang and Zhang 2018, Li et al. 2022, Lin et al. 2023).

**Figure 1.**
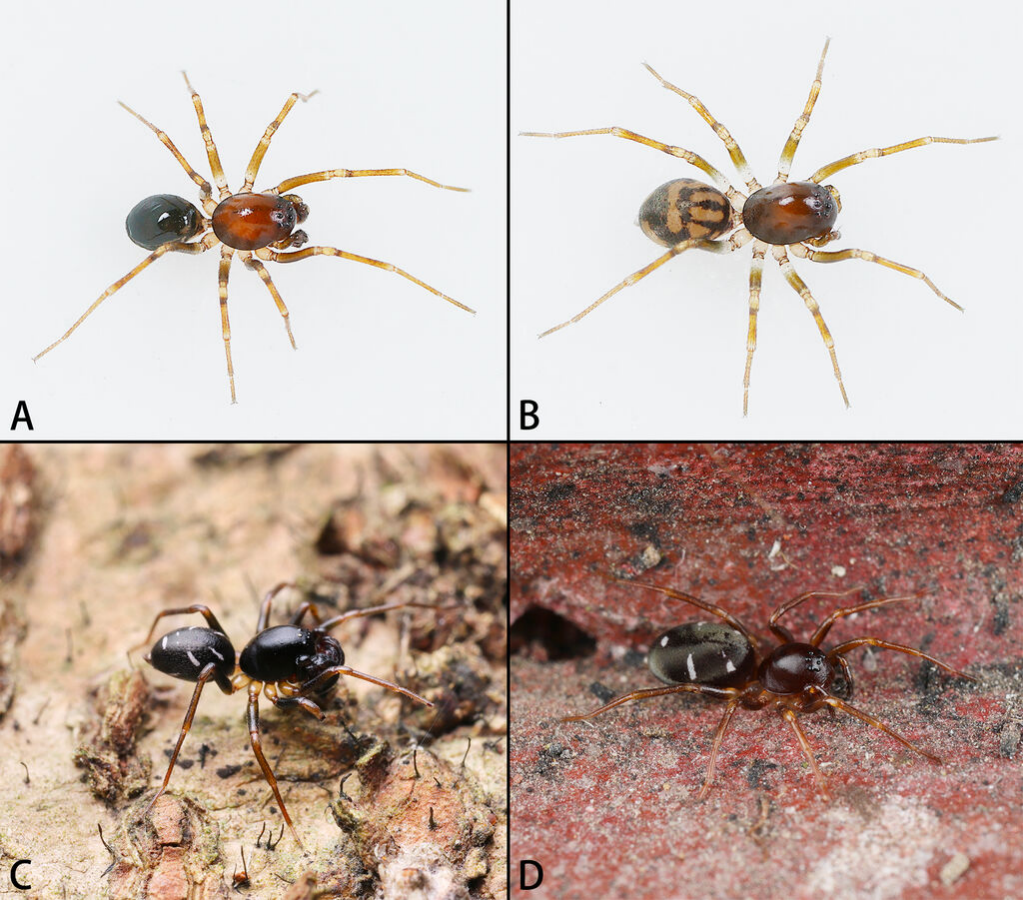
*Asceuahaocongi* sp. n. (**A**, **B**) and *A.zijin* sp. n. (**C**, **D**), alive. **A**, **C** Holotype male; **B**, **D** Paratype female. Photos by Fan Gao (**C**, **D**).

During the examination of spider collections from China (Hainan and Jiangsu), we found two new species and describe them here as *A.haocongi* sp. n. and *A.zijin* sp. n (Fig. 1). The goal of this paper is to provide descriptions of the new species.

## Materials and methods

All specimens were preserved in 80% ethanol. The spermathecae were cleared in trypsin enzyme solution to dissolve non-chitinous tissues. Specimens were examined under a Leica M205C stereomicroscope. Photomicrographs were taken with an Olympus C7070 zoom digital camera (7.1 megapixels). Laboratory habitus photographs were taken with a Sony A7RIV digital camera equipped with a Sony FE 90 mm Goss lens. Photos were stacked with Helicon Focus® (Version 7.6.1) or Zerene Stacker® (Version 1.04) and processed in Adobe Photoshop CC2022®. The distribution map was generated with ArcGIS v. 10.2 (ESRI Inc.).

All measurements are in millimetres (mm) and were obtained with an Olympus SZX16 stereomicroscope with a Zongyuan CCD industrial camera. All measurements of body lengths do not include the chelicerae. Eye sizes are measured as the maximum diameter from either the dorsal or frontal view. Leg measurements are given as follows: total length (femur, patella, tibia, metatarsus, tarsus). The type materials are deposited in the Institute of Zoology, Chinese Academy of Sciences in Beijing (**IZCAS**).

Abbreviations: **ALE**, anterior lateral eye; **AME**, anterior median eye; **C**, conductor; **CD**, copulatory duct; **CF**, cymbial furrow; **CO**, copulatory opening; **DTA**, dorsal tibial apophysis; **E**, embolus; **EB**, embolic base; **FD**, fertilisation duct; **H**, hood; **MOA**, median ocular area; **PLE**, posterior lateral eye; **PME**, posterior median eye; **RTA**, retrolateral tibial apophysis; **S**, spermatheca; **TA**, tegular apophysis; **VTA**, ventral tibial apophysis.

### Comparative material examined

*Asceuatorquata* (Simon, 1909), 4 males, without institution ID, China, Guangdong, Shenzhen, IZCAS.

## Taxon treatments

### Asceua haocongi

Lin & Li, 2023 sp. n.

8C2D0941-B907-5F08-928D-76B4E722CFE9

9067DEED-2DAB-4075-8E07-0392BD8D1EB3

#### Materials

- **Type status:** Holotype. **Occurrence:** recordedBy: Haocong Yang; sex: male; occurrenceID: 840AD147-8576-5FF5-9E4E-7454F1D069F4; **Location:** country: China; stateProvince: Hainan; locality: Sanya City, Jiyang District, Sanya Xiaodonghai Wedding Photography Base; verbatimElevation: 2 m; verbatimCoordinates: N18.2004°, E109.4984°; **Identification:** identifiedBy: Yejie Lin; **Event:** year: 2023; month: 2; day: 4; **Record Level:** institutionID: IZCAS-Ar44408
- **Type status:** Paratype. **Occurrence:** recordedBy: Haocong Yang; sex: female; occurrenceID: 0D741223-ED30-5E71-909A-A073F7FDF091; **Location:** country: China; stateProvince: Hainan; locality: Sanya City, Jiyang District, Sanya Xiaodonghai Wedding Photography Base; verbatimElevation: 2 m; verbatimCoordinates: N18.2004°, E109.4984°; **Identification:** identifiedBy: Yejie Lin; **Event:** year: 2023; month: 2; day: 4; **Record Level:** institutionID: IZCAS-Ar44409
- **Type status:** Paratype. **Occurrence:** recordedBy: Haocong Yang; sex: female; occurrenceID: 0D741223-ED30-5E71-909A-A073F7FDF091; **Location:** country: China; stateProvince: Hainan; locality: Sanya City, Jiyang District, Sanya Xiaodonghai Wedding Photography Base; verbatimElevation: 2 m; verbatimCoordinates: N18.2004°, E109.4984°; **Identification:** identifiedBy: Yejie Lin; **Event:** year: 2023; month: 2; day: 4; **Record Level:** institutionID: IZCAS-Ar44410
- **Type status:** Paratype. **Occurrence:** recordedBy: Haocong Yang; sex: female; occurrenceID: 0D741223-ED30-5E71-909A-A073F7FDF091; **Location:** country: China; stateProvince: Hainan; locality: Sanya City, Jiyang District, Sanya Xiaodonghai Wedding Photography Base; verbatimElevation: 2 m; verbatimCoordinates: N18.2004°, E109.4984°; **Identification:** identifiedBy: Yejie Lin; **Event:** year: 2023; month: 2; day: 4; **Record Level:** institutionID: IZCAS-Ar44411
- **Type status:** Paratype. **Occurrence:** recordedBy: Haocong Yang; sex: female; occurrenceID: 0D741223-ED30-5E71-909A-A073F7FDF091; **Location:** country: China; stateProvince: Hainan; locality: Sanya City, Jiyang District, Sanya Xiaodonghai Wedding Photography Base; verbatimElevation: 2 m; verbatimCoordinates: N18.2004°, E109.4984°; **Identification:** identifiedBy: Yejie Lin; **Event:** year: 2023; month: 2; day: 4; **Record Level:** institutionID: IZCAS-Ar44412
- **Type status:** Paratype. **Occurrence:** recordedBy: Haocong Yang; sex: female; occurrenceID: 0D741223-ED30-5E71-909A-A073F7FDF091; **Location:** country: China; stateProvince: Hainan; locality: Sanya City, Jiyang District, Sanya Xiaodonghai Wedding Photography Base; verbatimElevation: 2 m; verbatimCoordinates: N18.2004°, E109.4984°; **Identification:** identifiedBy: Yejie Lin; **Event:** year: 2023; month: 2; day: 4; **Record Level:** institutionID: IZCAS-Ar44413

#### Description

Male (holotype). Total length 2.13; carapace 1.07 long, 0.74 wide, opisthosoma 1.06 long, 0.71 wide. Eye sizes and interdistances: AME 0.08, ALE 0.06, PME 0.06, PLE 0.06, AME–AME 0.05, AME–ALE 0.03, PME–PME 0.08, PME–PLE 0.08, AME–PME 0.09, ALE–PLE 0.05. MOA 0.21 long, front width 0.19, back width 0.19. Clypeus height 0.29. Chelicerae with 2 promarginal teeth. Leg measurements: I 3.00 (0.76, 0.24, 0.73, 0.78, 0.49), II 2.34 (0.64, 0.25, 0.48, 0.61, 0.36), III 2.34 (0.64, 0.25, 0.47, 0.64, 0.36), IV 3.23 (0.84, 0.25, 0.73, 0.96, 0.45).

Colouration (Fig. 4A, B). Carapace red-brown, edge brown. Chelicerae, endites, labium and sternum red-brown. Legs yellow-brown with black ring pattern. Opisthosoma oval, dark brown with numbers of small yellow-brown spots, with ventral scutum covering opisthosoma, dorsal white with purple spots. Spinnerets purple.

**Figure 2.**
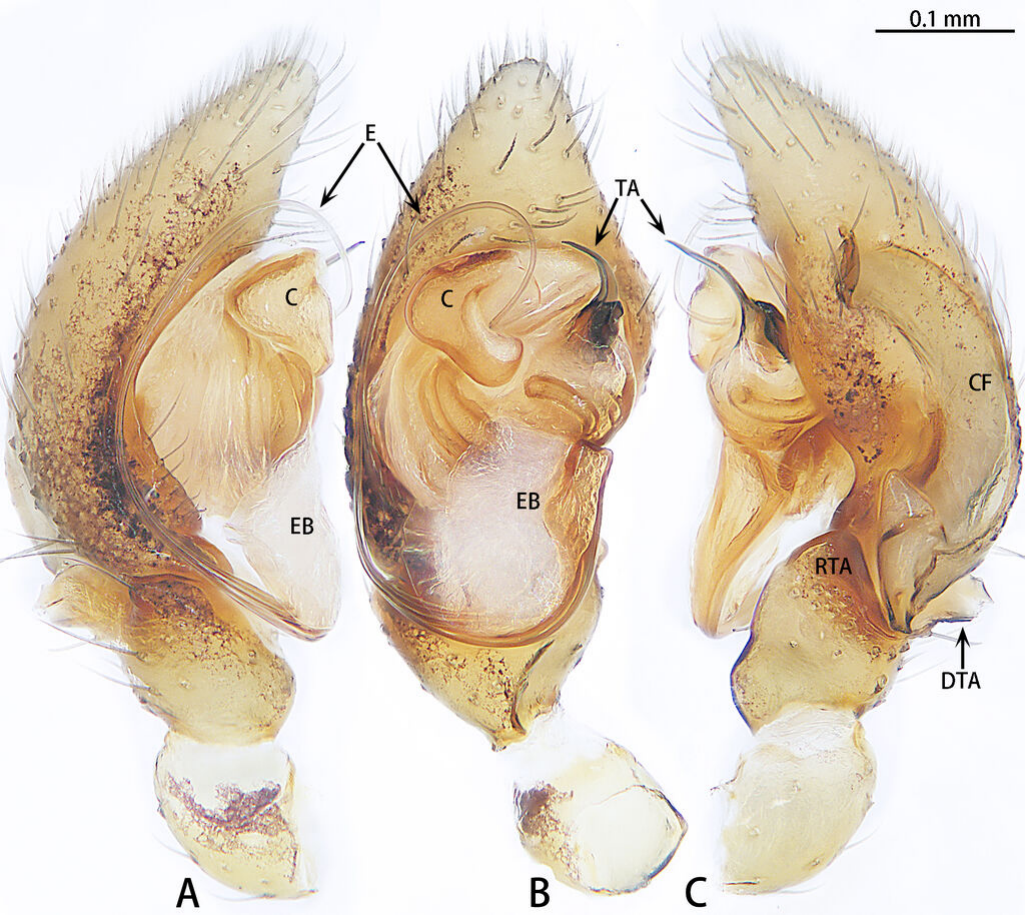
*Asceuahaocongi* sp. n., holotype male. **A** Prolateral view; **B** Ventral view; **C** Retrolateral view. Abbreviations: **C** conductor; **CF** cymbial furrow; **DTA** dorsal tibial apophysis; **E** embolus; **EB** embolic base; **RTA** retrolateral tibial apophysis; **TA** tegular apophysis.

Palp (Fig. 2). Patella almost as long as tibia. Tibia with two apophyses, retrolateral tibial apophysis blunt, triangular-shaped; dorsal tibial apophysis lamellate. Cymbium almost two times longer than wide. Cymbial furrow with a hood anteriorly and a digitiform apophysis posteriorly. Bulb almost 1.5 times longer than wide. Tegulum almost round. Tegular apophysis black, terminal sharp. Conductor bent, originating dorsally on bulb, with slightly widened tip. Embolus slender and whip-like, distal portion pointing to middle of conductor in ventral view. Base of embolus large, with large membranous prolateral part, arising at 7 o’clock position.

Female (IZCAS-Ar44409). Total length 2.57; carapace 1.22 long, 0.84 wide, opisthosoma 1.35 long, 0.92 wide. Eye sizes and interdistances: AME 0.08, ALE 0.07, PME 0.06, PLE 0.06, AME–AME 0.05, AME–ALE 0.03, PME–PME 0.09, PME–PLE 0.10, AME–PME 0.10, ALE–PLE 0.06. MOA 0.23 long, front width 0.20, back width 0.21. Clypeus height 0.33. Chelicerae with 2 promarginal teeth. Leg measurements: I 2.90 (0.77, 0.27, 0.65, 0.73, 0.48), II 2.44 (0.67, 0.27, 0.49, 0.61, 0.40), III 2.45 (0.66, 0.28, 0.49, 0.66, 0.36), IV 3.34 (0.87, 0.28, 0.75, 0.98, 0.46).

Colouration (Fig. 4C, D). As in male, except lacking scutum on opisthosoma and opisthosoma with three pairs of white spots, first pair oblong-oval, longitudinal, largest; second pair irregular in shape, positioned lateral to the first pair, third pair ovoid, transverse, slightly connected to the second pair.

**Figure 3.**
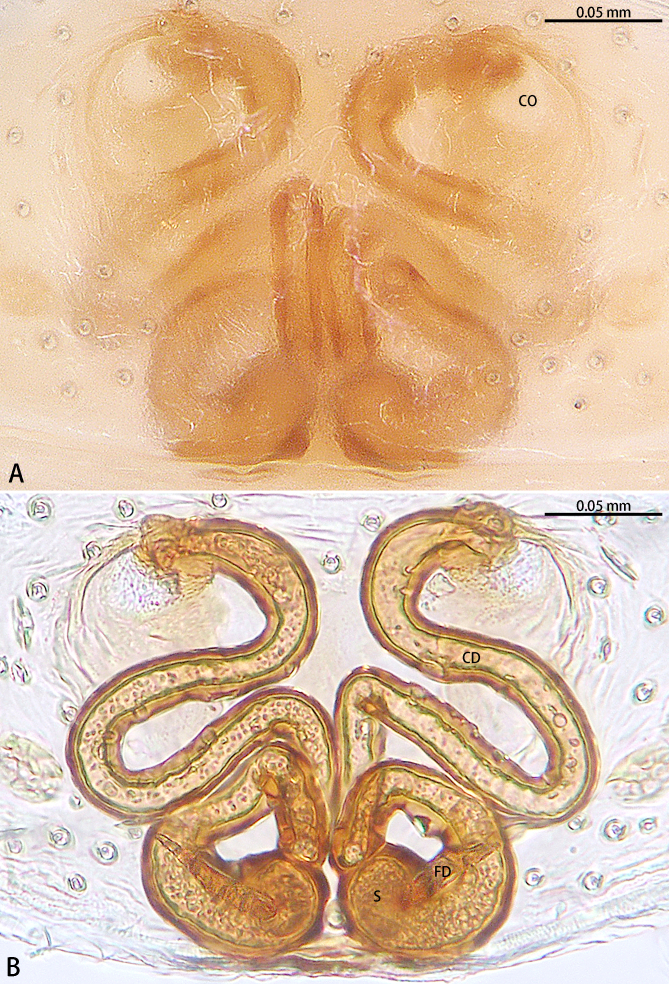
*Asceuahaocongi* sp. n., paratype female. **A** Epigyne, ventral view; **B** Vulva, dorsal view. Abbreviations: **CD** copulatory duct; **CO** copulatory opening; **FD** fertilisation duct; **S** spermatheca.

**Figure 4.**
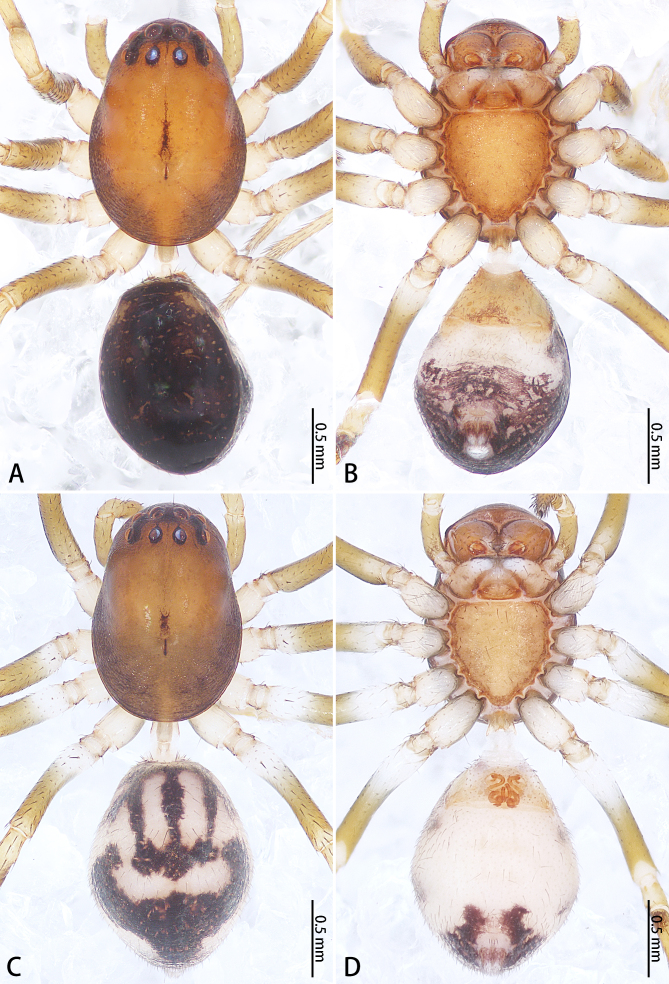
*Asceuahaocongi* sp. n., male holotype (**A**, **B**) and female paratype (**C**, **D**), Habitus. **A**, **C** Dorsal view; **B**, **D** Ventral view.

Epigyne (Fig. 3). Epigynal plate as long as wide. Copulatory openings located on anterior portion of epigyne. Copulatory ducts intertwined. Spermathecae kidney-shaped, two times wider than copulatory ducts. Fertilisation ducts directed at the 2 o'clock position from spermathecae.

#### Diagnosis

The male is similar to that of *Asceuaradiosa* Jocqué, 1986 by the triangular-shaped retrolateral tibial apophysis, same shaped cymbial furrow and tegular apophysis needle-shaped. Females share the same shape of the copulatory duct. However, the new species can be distinguished from *A.radiosa* by the cymbial furrow with a digitiform apophysis posteriorly (Fig. 2C) [vs. absent in *A.radiosa* (see Jocqué (1986), fig. 4)] and tegular apophysis strongly curved (Fig. 2B) [vs. almost straight in *A.radiosa* (see Jocqué (1986), fig. 5)]. The female can be distinguished from *A.radiosa* by the kidney-shaped spermatheca (Fig. 3B) [vs. oval in *A.radiosa* (see Jocqué (1986), fig. 8)].

#### Etymology

The species is named after the collector Mr. Haochong Yang; noun (name) in genitive case.

#### Distribution

Known only from the type locality (Fig. 9).

### Asceua zijin

Lin & Li, 2023 sp. n.

2EAC2867-CC35-5E67-B804-8888B4F7D92A

A2DFE36D-A028-4C42-ADDE-C7D39592C7E1

#### Materials

- **Type status:** Holotype. **Occurrence:** recordedBy: Fan Gao; sex: male; occurrenceID: D22DAF21-8C47-5E82-B8BC-DDBD8CC8ACBC; **Location:** country: China; stateProvince: Jiangsu; locality: Nanjing City, Xuanwu District, Zijin Mountain; verbatimElevation: 75 m; verbatimCoordinates: N32.0794°, E118.8298°; **Identification:** identifiedBy: Yejie Lin; **Event:** year: 2019; month: 12; day: 13; **Record Level:** institutionID: IZCAS-Ar44414
- **Type status:** Paratype. **Occurrence:** recordedBy: Fan Gao; sex: female; occurrenceID: 989B35C2-6891-546B-A70D-ACD332C95103; **Location:** country: China; stateProvince: Jiangsu; locality: Nanjing City, Xuanwu District, Zijin Mountain; verbatimElevation: 75 m; verbatimCoordinates: N32.0794°, E118.8298°; **Identification:** identifiedBy: Yejie Lin; **Event:** year: 2019; month: 12; day: 13; **Record Level:** institutionID: IZCAS-Ar44415

#### Description

Male (holotype). Total length 3.33; carapace 1.56 long, 1.17 wide, opisthosoma 1.77 long, 1.03 wide. Eye sizes and interdistances: AME 0.07, ALE 0.06, PME 0.07, PLE 0.07, AME–AME 0.06, AME–ALE 0.06, PME–PME 0.09, PME–PLE 0.14, AME–PME 0.13, ALE–PLE 0.05. MOA 0.26 long, front width 0.17, back width 0.22. Clypeus height 0.40. Chelicerae with 2 promarginal teeth. Leg measurements: I 4.12 (1.06, 0.37, 1.01, 1.03, 0.65), II 3.69 (0.99, 0.38, 0.84, 0.91, 0.57), III 3.41 (0.96, 0.41, 0.70, 0.88, 0.46), IV 4.38 (1.17, 0.42, 1.06, 1.18, 0.55).

Colouration (Fig. 8A, B). Carapace brown, middle with two oblong black-brown spots. Chelicerae, endites, labium and sternum brown. Legs yellow-brown. Opisthosoma oval, black, dorsum with three pairs of pale oblong spots, second one largest, followed by a pale spot. Spinnerets black.

**Figure 5.**
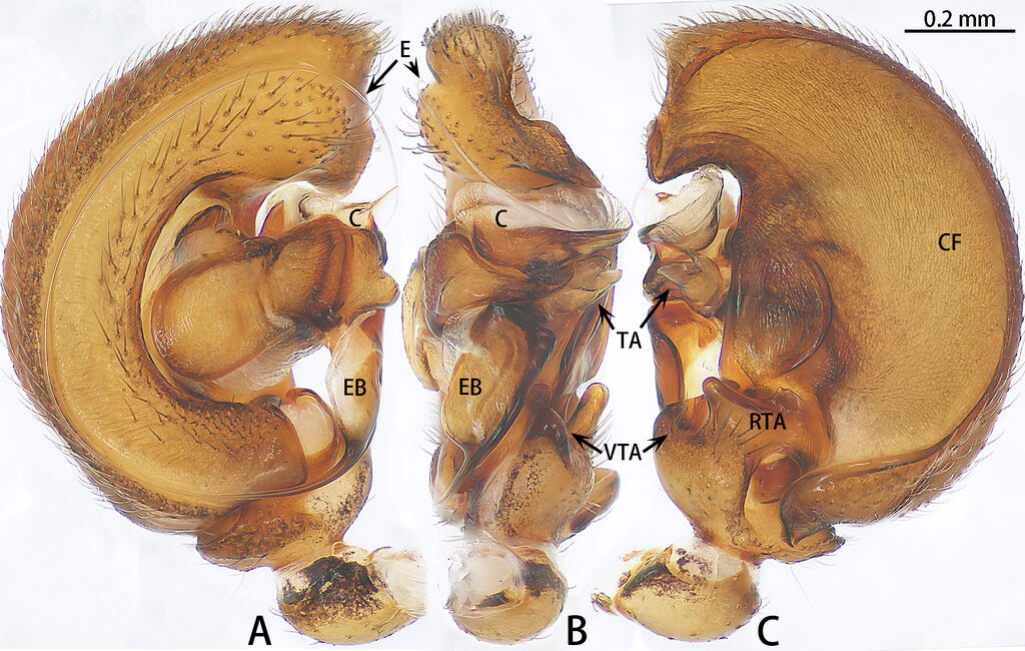
*Asceuazijin* sp. n., holotype male. **A** Prolateral view; **B** Ventral view; **C** Retrolateral view. Abbreviations: **C** conductor; **CF** cymbial furrow; **E** embolus; **EB** embolic base; **RTA** retrolateral tibial apophysis; **TA** tegular apophysis; **VTA** ventral tibial apophysis.

Palp (Figs 5, 6b). Tibia two times longer than patella. Tibia with two apophyses; ventral tibial apophysis digitiform; retrolateral tibial apophysis bifurcate, ventral ramus digitiform, transverse, dorsal ramus sickle-shaped. Cymbium almost 1.5 times longer than wide. Cymbial furrow wide, almost as wide as cymbium in retrolateral view. Tegulum almost oblong. Tegular apophysis with two lateral processes. Conductor membranous, with a prolateral apophysis. Embolus slender and whip-like. Base of embolus large, with membranous prolateral part, arising at the 6:30 o'clock position.

Female (IZCAS-Ar44415). Total length 3.98; carapace 1.70 long, 1.16 wide, opisthosoma 2.28 long, 1.38 wide. Eye sizes and interdistances: AME 0.06, ALE 0.08, PME 0.07, PLE 0.08, AME–AME 0.06, AME–ALE 0.08, PME–PME 0.09, PME–PLE 0.15, AME–PME 0.15, ALE–PLE 0.04. MOA 0.27 long, front width 0.16, back width 0.23. Clypeus height 0.42. Chelicerae with 2 promarginal teeth. Leg measurements: I 3.70 (1.00, 0.39, 0.86, 0.85, 0.60), II 3.45 (0.95, 0.41, 0.75, 0.80, 0.54), III 3.40 (0.93, 0.44, 0.70, 0.88, 0.45), IV 4.23 (1.10, 0.44, 1.00, 1.12, 0.57).

Colouration (Fig. 8C, D). Similar to that of male, except paler.

**Figure 7.**
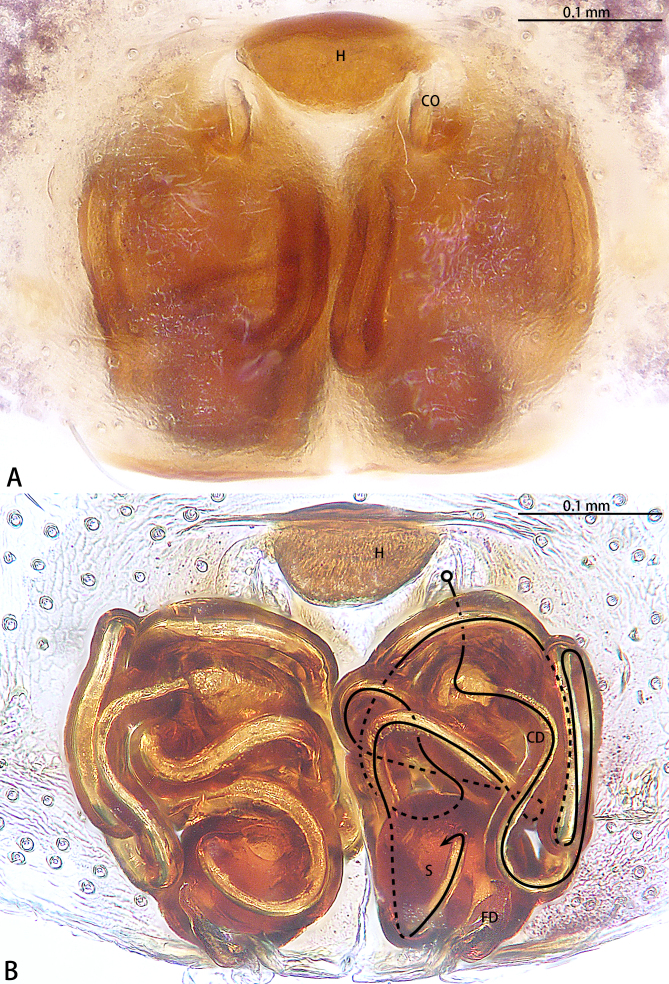
*Asceuazijin* sp. n., paratype female. **A** Epigyne, ventral view; **B** Vulva, dorsal view. Abbreviations: **CD** copulatory duct; **CO** copulatory opening; **FD** fertilisation duct; **H** hood; **S** spermatheca.

Epigyne (Fig. 7). Epigynal plate as long as wide, with a hood anteriorly, the hood almost 1.5 times longer than wide. Copulatory openings located on anterior portion of epigyne. Copulatory ducts intertwined, starting part expanded. Spermathecae oval. Fertilisation ducts directed at the 1:00 o'clock position from spermathecae.

#### Diagnosis

**Figure 8.**
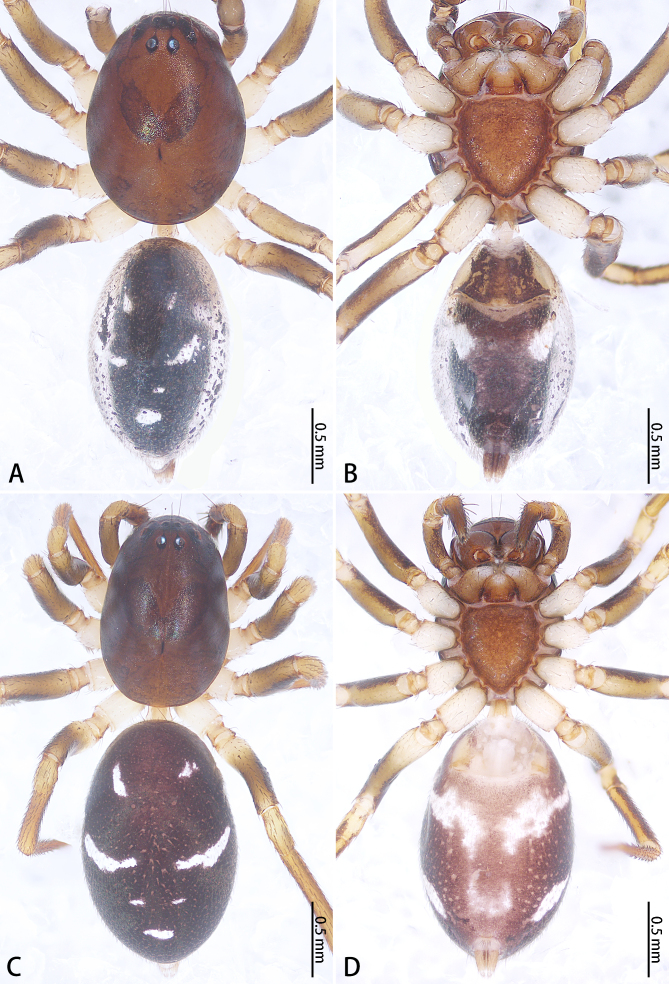
*Asceuazijin* sp. n., male holotype (**A**, **B**) and female paratype (**C**, **D**), Habitus. **A**, **C** Dorsal view; **B**, **D** Ventral view.

The male is similar to *Asceuatorquata* (Simon, 1909) and *A.japonica* (Bösenberg & Strand, 1906) by the same shape of retrolateral tibial apophysis and cymbium, long and slender embolus. Females share the same shape of the copulatory duct. However, the new species can be distinguished from *A.torquata* by the opisthosoma dorsum with seven pale spots (Fig. 8) [vs. four yellow spots, a triangular black spot in the middle of the largest spot (see Zhang and Zhang (2018), figs. 7A, C)] conductor with an apophysis prolaterally and tegular apophysis with a lamellate apophysis (Fig. 6b) [vs. absent in *A.torquata* (Fig. 6a)]. The female can be distinguished from *A.torquata* and *A.japonica* by the obvious, semi-circular hood, 1.5 times longer than wide (Fig. 7B) [vs. oval in *A.torquata* (see Zhang and Zhang (2018), fig. 8D) and eight times longer than wide in *A.japonica* (see Ono (2009), fig. 30)] and copulatory ducts starting part expanded (Fig. 7B) [vs. absent in *A.japonica* (see Ono (2009), fig. 30)].

#### Etymology

The specific name refers to the type locality; noun (name) in genitive case.

#### Distribution

**Figure 9.**
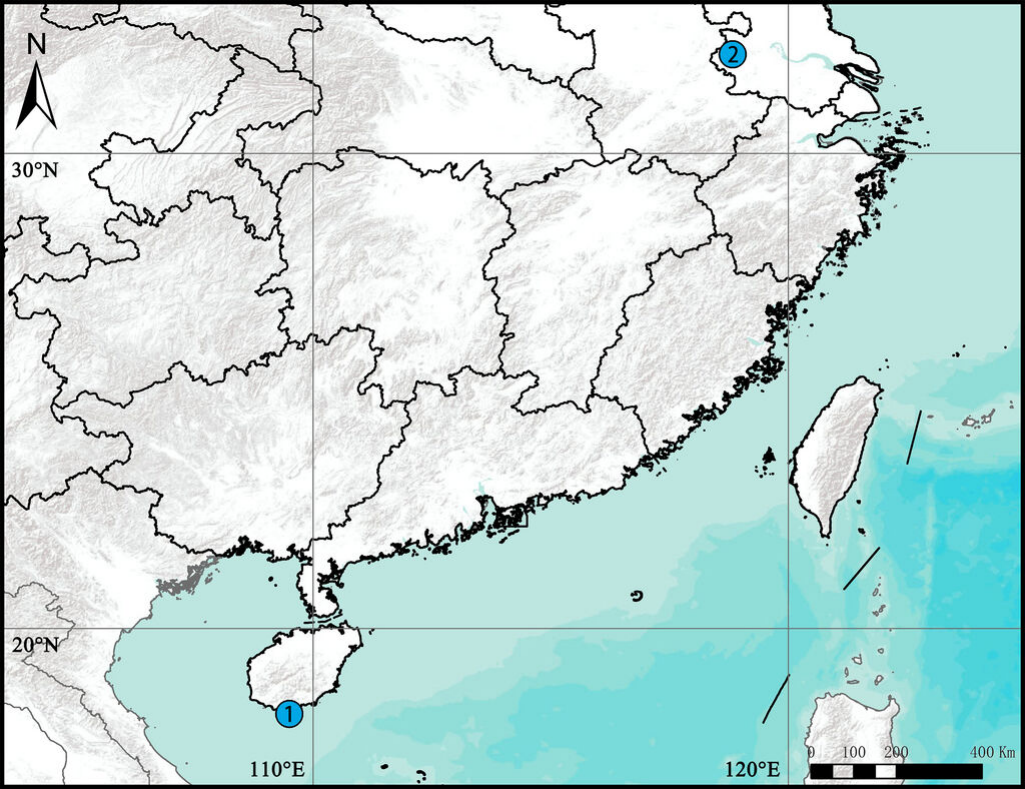
Distribution records of new *Asceua* species in China: **1** *A.haocongi* sp. n.; **2** *A.zijin* sp. n.

Known only from the type locality (Fig. 9).

## Supplementary Material

XML Treatment for Asceua haocongi

XML Treatment for Asceua zijin

## Figures and Tables

**Figure 6a.**
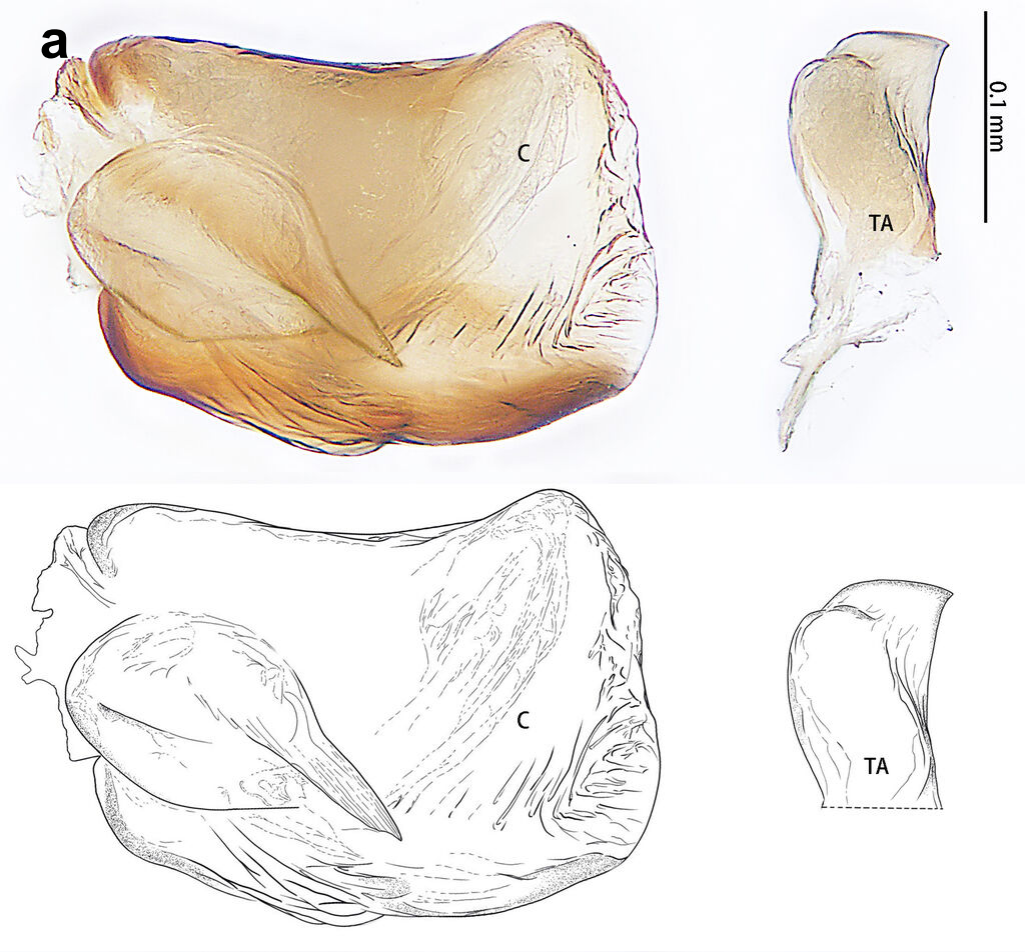
A.torquata

**Figure 6b.**
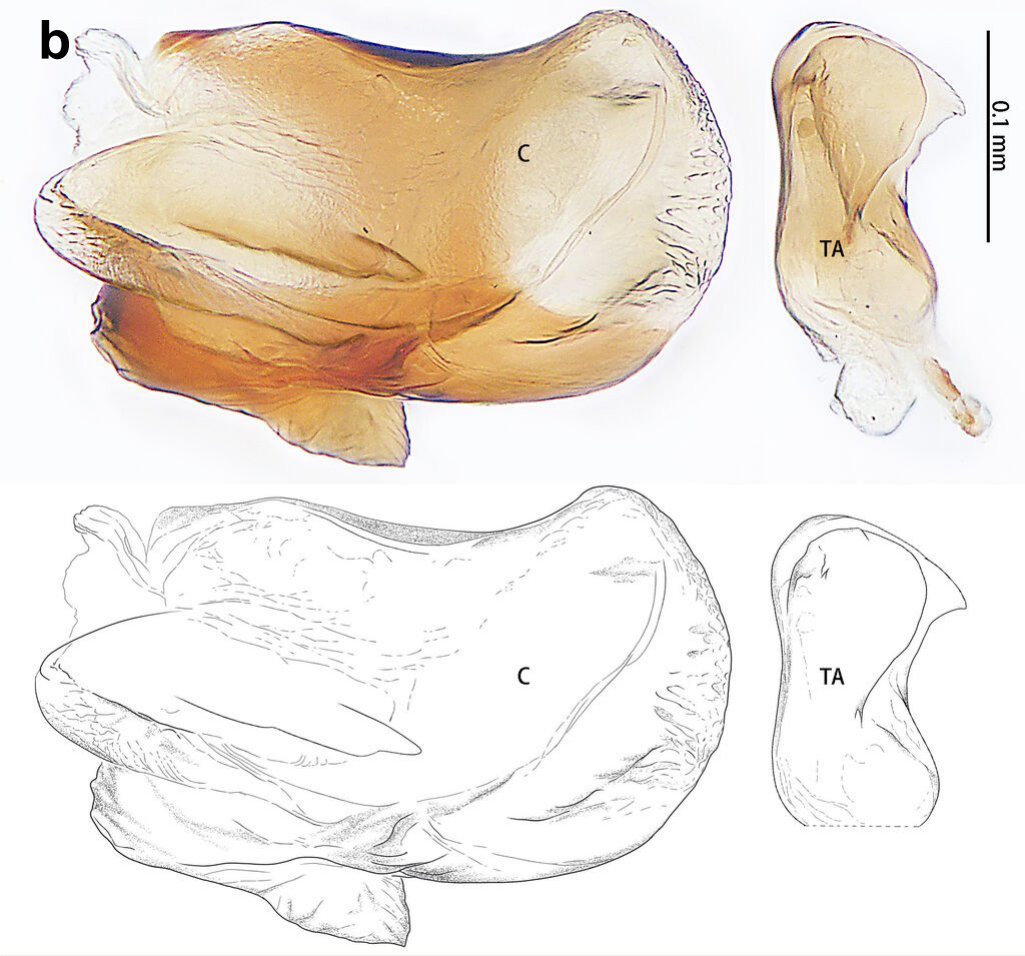
*A.zijin* sp. n.
